# Preoperative depressive symptoms as a marker of postoperative delirium risk in older adults: a prospective cohort study in spine surgery

**DOI:** 10.3389/fmed.2026.1936636

**Published:** 2026-09-15

**Authors:** Yannick Veser, Jonas Müller, Angelika Fleischmann, Sophie Leroy, Johanna Ruhnau, Jan-Uwe Müller, Henry W. S. Schröder, Agnes Flöel, Robert Fleischmann

**Affiliations:** 1 Department of Neurology, University Medicine Greifswald, Greifswald, Germany; 2 Department of Neurosurgery, University Medicine Greifswald, Greifswald, Germany; 3 Department of Neurology, Charité – University Medicine Berlin, Berlin, Germany

**Keywords:** delirium, depression, pain, postoperative neurocognitive disoders, spine surgery, geriatrics, older adults

## Abstract

**Introduction:**

Postoperative delirium (POD) is a frequent perioperative complication in older adults and is associated with delayed recovery and adverse cognitive outcomes. We investigated whether preoperative depressive and anxiety symptoms were associated with POD in adults aged 60 years or older undergoing elective spine surgery.

**Methods:**

This prospective cohort analysis was conducted within the Cognitive Dysfunction Following Elective Spine Surgery in Older Patients (CONFESS) study. Depressive and anxiety symptoms were assessed preoperatively using the Hospital Anxiety and Depression Scale. POD was screened using the Nursing Delirium Screening Scale and confirmed by a physician according to DSM-5 criteria.

**Results:**

Of 124 enrolled patients, 99 participants underwent surgery and 93 had complete data for the present analysis (mean age 71.2 ± 7.1 years; 45 women). Sixteen patients (17.2%) developed POD. Each one-point increase in the depression subscale score was associated with higher odds of POD (odds ratio 1.29, 95% confidence interval 1.06–1.55; *p* = 0.01). A depression subscale score of 8 or higher was associated with sixfold higher odds of POD (odds ratio 6.00, 95% confidence interval 1.86–19.32; *p* < 0.01). The continuous association remained significant after adjustment for age, sex, and preoperative cognitive performance (adjusted odds ratio 1.25, 95% confidence interval 1.03–1.52; *p* = 0.03). Anxiety symptoms were not significantly associated with POD.

**Discussion:**

Preoperative depressive symptom burden was associated with POD after adjustment for age, sex, and preoperative cognitive performance. Given the limited number of POD events, these findings should be interpreted cautiously but may support further evaluation of depressive symptom screening as an adjunct to perioperative geriatric risk stratification.

**Clinical Trial registration:**

This study was prospectively registered on 1 March 2018 via ClinicalTrials.gov (ID: NCT03486288).

## 1 Introduction

The world's older population continues to grow, with the number of people aged 60 years and older projected to rise from around 900 million in 2015 to 2 billion by 2050 (1). This demographic shift is of great importance for health care systems, as it parallels an increase in the number of surgical interventions in older adults, reflecting both their higher prevalence of musculoskeletal disease and improved treatment options (2). Spine surgery is particularly affected by this development. Globally, Australia exhibited a 30% increase in lumbar spine surgery between 2003 and 2013, Norway saw a threefold rise in lumbar stenosis surgery from 1999 to 2013, and anterior cervical discectomy and fusion increased eightfold in the United States between 1990 and 2004 (3–5). Despite major advances in surgical and anesthetic care, elderly patients remain at an elevated risk for postoperative complications that critically affect recovery (6). One of the most common and clinically significant is postoperative delirium (POD) (7). According to the fifth edition of the Diagnostic and Statistical Manual of Mental Disorders (DSM-5), delirium is defined as a disturbance in attention and awareness that develops over a short period of time, fluctuates throughout the day, and is accompanied by an additional cognitive disturbance, which cannot be better explained by a preexisting neurocognitive disorder (8).

Preoperative patient factors are well-established predictors of POD. Non-modifiable factors include age, prior neurologic disease (e.g., dementia, stroke, previous delirium), and multimorbidity. In contrast, potentially modifiable or clinically addressable preoperative factors may provide additional opportunities for risk stratification and prevention, yet remain comparatively understudied. Depressive symptom burden is of particular interest in this context. Depression affected 4.4% of the world's population in 2015, with prevalence estimates of approximately 8% among older adults (9). Depression has been shown to exacerbate postoperative pain, impair wound healing and mobilization, and prolong hospital stay (10–12). Furthermore, several studies suggest that preoperative depression may independently increase the risk of POD (13–15). However, most of these studies relied on diagnosed depressive disorders, leaving the contribution of subclinical affective symptoms largely unexplored.

The pathophysiological overlap between depression and delirium may involve shared mechanisms of neuroinflammation, cytokine activation, and network dysfunction. Elevated circulating interleukin-6 (IL-6) levels are consistently associated with both depressive symptoms and postoperative delirium (16, 17). These inflammatory changes, in conjunction with hypothalamic–pituitary–adrenal (HPA) axis dysregulation and reduced neurotrophic signaling, can impair cognitive resilience and synaptic plasticity in the aging brain (18). These shared mechanisms provide a biological rationale for investigating depressive symptom burden as a potential marker of perioperative neurocognitive vulnerability in older adults.

The incidence of POD varies widely by surgical type, ranging from approximately 4% in cataract surgery to over 50% in abdominal aortic aneurysm repair or orthopedic procedures (19, 20). This heterogeneity emphasizes the need for surgery-specific analyses. In spine surgery, few studies have systematically examined the relationship between depressive symptoms, particularly subclinical forms, and POD (21–23). Moreover, the impact of coexisting anxiety symptoms remains poorly understood, despite their high prevalence in patients facing major surgery.

We hypothesized that higher preoperative depressive and anxiety symptom burden, assessed independently of a diagnosed affective disorder, would be associated with greater odds of postoperative delirium after elective spine surgery in older adults. We further hypothesized a graded association between depressive symptom burden and POD. This prospective, procedure-specific analysis aimed to evaluate depressive symptoms as a clinically feasible marker of perioperative vulnerability and risk stratification.

## 2 Materials and methods

### 2.1 Study registration, ethics, and reporting

This prospective cohort analysis was conducted within the Cognitive Dysfunction Following Elective Spine Surgery in Older Patients (CONFESS) study, which was jointly performed by the Departments of Neurology and Neurosurgery in cooperation with the Department of Anesthesiology at University Medicine Greifswald, Germany (24). The study was prospectively registered at ClinicalTrials.gov on 1 March 2018 (NCT03486288).

The study was conducted in accordance with the Declaration of Helsinki. The study protocol was approved by the Ethics Committee of University Medicine Greifswald on 16 February 2018 (approval number BB 192/17). All participants provided written informed consent before study participation.

This study is reported in accordance with the Strengthening the Reporting of Observational Studies in Epidemiology (STROBE) statement for cohort studies.

### 2.2 Study design and patient population

Patients were recruited between March 2018 and March 2020 through the inpatient and outpatient services of the Department of Neurosurgery. Eligible patients were aged 60 years or older, scheduled for elective spine surgery without opening of the dura, native German speakers, and able to provide informed consent independently. Exclusion criteria were a documented diagnosis of dementia, a chronic neurodegenerative disorder, a psychiatric disorder with psychotic symptoms, treatment with centrally acting medication including antidepressants, antipsychotics, sedatives, or alpha-1-receptor antagonists, participation in an interventional study, displaceable metallic or electronic implants, active neoplastic disease, or inability to attend the planned follow-up visits. Eligibility screening was performed by experienced neurosurgeons as part of routine clinical care. The present analysis included participants with available preoperative HADS data and postoperative delirium assessments. The present study therefore represents a secondary analysis of the prospectively recruited CONFESS cohort. No separate *a priori* sample-size calculation was performed for the association between preoperative HADS scores and postoperative delirium. The analytic sample size was therefore determined by the number of participants with available preoperative HADS data and postoperative delirium assessments. Given the limited number of POD events available for HADS-D modeling, effect estimates are reported with 95% confidence intervals and interpreted cautiously.

### 2.3 Assessment of depression, anxiety and delirium

The baseline examination was performed up to 14 days before the scheduled surgery by trained study personnel, including briefed medical students and study nurses. Most baseline visits took place one to two days prior to surgery.

Baseline assessment included, among other measures, the evaluation of depressive and anxiety symptoms using the Hospital Anxiety and Depression Scale (HADS). This self-report questionnaire consists of 14 items, with seven items assessing anxiety (HADS-A) and seven items assessing depression (HADS-D). It was developed by Zigmond and Snaith in 1983 (25). The established cut-off scores for the individual subscales are 8 points for mild, 11 for moderate, and 15 for severe symptom levels, with anxiety and depression scored separately (25, 26).

The HADS demonstrates high internal consistency (Cronbach's alpha 0.82) and good sensitivity and specificity, both ranging from 0.7 to 0.9. Its performance is largely independent of sex or patient population, whether psychiatric, somatic, or general (27). Importantly, the HADS minimizes confounding by excluding somatic items that could overlap with physical illness, making it suitable for use in medically compromised populations. It has also been validated for use in elderly individuals and patients with back pain (28).

To detect delirium, a standardized three-step approach was applied. Screening was performed once before surgery during baseline assessment and then repeatedly after surgery, starting four hours postoperatively. From the first postoperative day onward, delirium screening was conducted twice daily on working days by briefed physicians during morning and day shifts, and by trained nursing staff during night shifts and weekends as part of routine clinical care.

Delirium screening was performed using the Nursing Delirium Screening Scale (Nu-DESC). The Nu-DESC consists of five items assessing disorientation, inappropriate behavior, inappropriate communication, illusions or hallucinations, and psychomotor retardation (29). This tool was selected because of its brevity, ease of application, and the fact that no special training is required for its reliable use. The Nu-DESC has demonstrated excellent interrater reliability, with sensitivity and specificity of 98% and 92%, respectively, when applying a cut-off score of ≥ 2 (30–32).

To enhance diagnostic accuracy, positive Nu-DESC screenings were followed by a physician assessment according to DSM-5 criteria (33). If delirium was detected, screening continued for up to one week postoperatively. In the absence of delirium, days three to five were covered by systematic chart review, including entries from both medical and nursing documentation.

### 2.4 Secondary endpoint measures

To evaluate potential changes in depressive or anxiety symptoms after surgery, the HADS was reassessed three months postoperatively during a follow-up screening. Patients were invited for this assessment in conjunction with their routine neurosurgical control appointment to minimize additional burden.

To determine whether depressive and anxiety symptoms were influenced by back pain, we included a specific and widely used patient-reported outcome measure for spinal conditions, the Oswestry Disability Index (ODI). The ODI consists of ten items that address limitations in daily activities such as personal hygiene, walking, sitting, and sleeping. Each item offers six response options ranging from 0 (no impairment) to 5 (maximum impairment) (34, 35). An overall score of ≤ 22 points is considered indicative of an acceptable symptom state (36). The ODI was administered both at baseline and at the three-month follow-up.

To assess postoperative pain, a numeric rating scale was used. Pain intensity was recorded four hours after surgery and subsequently during morning and day shifts on the following days, continuing until the resolution of delirium or hospital discharge. Additionally, the total length of hospital stay was obtained from the official medical record after patient discharge.

### 2.5 Statistical analysis

Data were collected and managed in pseudonymized form using CentraXX Trial (Kairos GmbH, Bochum, Germany) and analyzed using IBM SPSS Statistics, version 25 (IBM, Armonk, NY, United States). Continuous variables are presented as mean ± standard deviation, and categorical variables as number and percentage. Effect estimates are reported with 95% confidence intervals. Statistical significance was set at *p* < 0.05. The primary analyses used a complete-case approach. Six of the 99 surgical participants did not provide a preoperative HADS-D score because they declined the HADS assessment; all six subsequently developed POD. These participants were therefore excluded from HADS-D analyses. Because missing HADS-D data were associated with POD status, the missingness cannot be considered completely at random. No imputation of continuous HADS-D values was performed. For the dichotomous HADS-D analysis (<8 vs. ≥ 8), we performed an extreme-case sensitivity analysis by assigning all six missing POD cases first to the HADS-D < 8 category and then to the HADS-D ≥ 8 category. Secondary analyses were based on complete observations for the variables included in the respective analysis.

Binomial logistic regression analyses were performed to examine the associations of preoperative depressive and anxiety symptoms with POD. HADS-A and HADS-D scores were analyzed as continuous variables and as dichotomous variables using a score of 8 as the threshold for clinically relevant symptoms. Model fit was evaluated using the omnibus test of model coefficients. Regression results are reported as odds ratios with 95% confidence intervals. Independent-samples t-tests were used to compare mean HADS-A and HADS-D scores between patients with and without POD. Separate linear regression analyses were performed to examine the associations of the Oswestry Disability Index with HADS-A and HADS-D scores. The association between the Oswestry Disability Index and POD was examined using binomial logistic regression. An adjusted logistic regression model was used to evaluate the association between continuous HADS-D scores and POD after adjustment for age, sex, and the preoperative mean Consortium to Establish a Registry for Alzheimer's Disease score. Associations of depressive symptom burden with postoperative pain and length of hospital stay were examined using linear regression models. Normality and homogeneity of variance were assessed before parametric analyses. Where applicable, Mann–Whitney U or Kruskal–Wallis tests were performed as sensitivity analyses and yielded findings consistent with the primary analyses.

## 3 Results

### 3.1 Patient description and delirium occurrence

Over the two-year recruitment period, 565 patients were screened for study eligibility and 124 consented to participate in the CONFESS study. Of these, 25 did not undergo the planned eligible surgery because consent was subsequently withdrawn or the planned procedure was cancelled, leaving 99 participants who underwent surgery. POD occurred in 22 of these 99 surgical participants (22.2%). Six of the 22 patients who developed POD had declined or not completed the preoperative HADS assessment for other reasons, and therefore had no HADS-D score available. These patients could not be included in the HADS-D analyses. The final complete-case HADS-D cohort therefore comprised 93 participants, including 16 with POD and 77 without POD. The study population comprised 45 women (mean age 72.5 ± 6.3 years) and 48 men (mean age 70.0 ± 7.6 years). Baseline characteristics of the complete-case cohort according to postoperative delirium status are presented in Table 1. Participant flow is summarized in Supplementary Figure S1.

**Table 1:** Selected baseline characteristics according to postoperative delirium status in the complete-case HADS-D cohort.

Characteristic	No POD (*n* = 77)	POD (*n* = 16)	Total (*n* = 93)
Age, years, mean ± SD	70.51 ± 7.15	74.75 ± 5.59	71.24 ± 7.07
**Sex, *n* (%)**			
Male	40 (51.9)	8 (50.0)	48 (51.6)
Female	37 (48.1)	8 (50.0)	45 (48.4)
**Preoperative depressive symptoms, *n* (%)**			
HADS-D < 8	66 (85.7)	8 (50.0)	74 (79.6)
HADS-D ≥ 8	11 (14.3)	8 (50.0)	19 (20.4)

Data are presented as mean ± standard deviation or *n* (%). Percentages are column percentages. HADS-D, depression subscale of the Hospital Anxiety and Depression Scale; POD, postoperative delirium; SD, standard deviation.

### 3.2 Effect of depressive and anxiety symptoms on POD

Nineteen participants (20.4%) had a preoperative HADS-D score of 8 or higher, whereas 74 participants had a HADS-D score below 8. Sixteen participants had a HADS-A score of 8 or higher, and 77 had a HADS-A score below 8. The binomial logistic regression model assessing the effect of HADS scores on the occurrence of POD was statistically significant, *χ*^2^(2) = 10.56, *p* = 0.01. Higher HADS-D scores were associated with greater odds of POD, with an odds ratio of 1.29 (95% CI 1.06–1.55) per one-point increase, *p* = 0.01. Using the established cut-off of 8 points, a HADS-D score ≥8 was associated with sixfold higher odds of POD (odds ratio 6.00, 95% CI 1.86–19.32, *p* < 0.01). Because all six participants with missing HADS-D data developed POD, an extreme-case sensitivity analysis was performed for the dichotomous HADS-D comparison. Assigning all six missing POD cases to the HADS-D < 8 category yielded an odds ratio of 3.43 (95% CI 1.17–10.08), whereas assigning all six to the HADS-D ≥ 8 category yielded an odds ratio of 10.50 (95% CI 3.57–30.86). Thus, the positive association between HADS-D ≥ 8 and POD persisted under both extreme allocations. Mean HADS-D scores were higher in patients who developed POD (7.44 ± 4.97) than in those who did not (4.25 ± 2.70), with a mean difference of 3.19 [95% CI 0.49–5.89; t(16.89) = 2.50, *p* = 0.02]. In the adjusted logistic regression model, each one-point increase in HADS-D remained associated with higher odds of POD after adjustment for age, sex, and preoperative cognitive performance (adjusted odds ratio 1.25, 95% CI 1.03–1.52; *p* = 0.03).

Among the 93 participants with available HADS-D data, 8 of 19 participants (42.1%) with a HADS-D score ≥8 developed POD, compared with 8 of 74 participants (10.8%) with a HADS-D score <8 (Figure 1). Conversely, 66 of 74 participants (89.2%) with a HADS-D score <8 remained free of POD. Within this complete-case cohort, HADS-D demonstrated a sensitivity of 50.0%, a specificity of 85.7%, a positive predictive value of 42.1%, and a negative predictive value of 89.2%. These diagnostic performance estimates should be interpreted cautiously because HADS-D data were unavailable for six additional surgical participants, all of whom developed POD. In contrast, HADS-A scores showed neither a significant group difference nor a significant association with the occurrence of POD. Furthermore, no significant change in HADS-A or HADS-D scores was observed between the preoperative and postoperative assessments.

**Figure 1:**
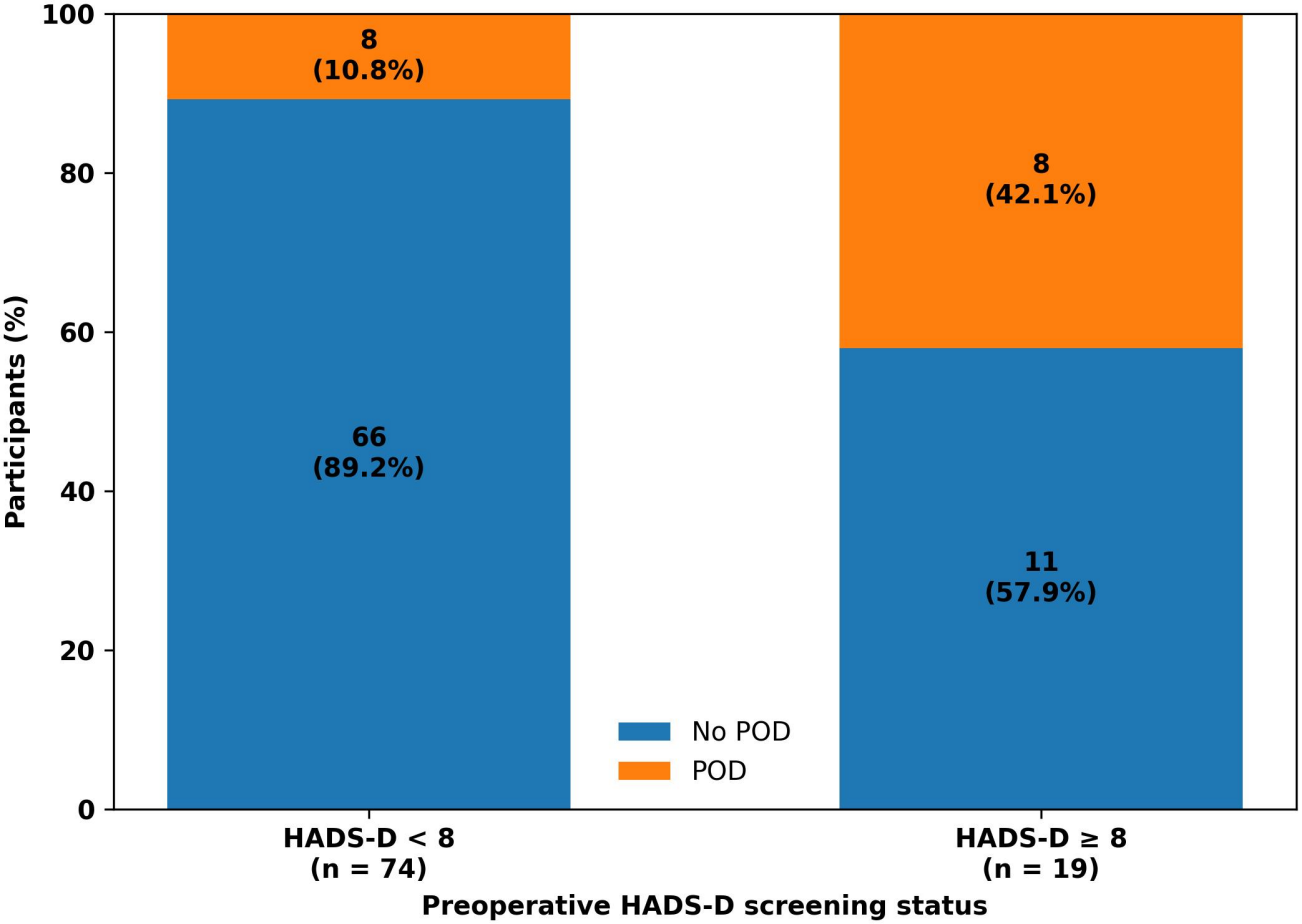
**Postoperative delirium according to preoperative HADS,D screening status.** Among 93 participants with available preoperative HADS-D data, POD occurred in 8 of 74 participants (10.8%) with a HADS-D score <8 and in 8 of 19 participants (42.1%) with a HADS-D score ≥8. Six additional surgical participants who developed POD did not provide preoperative HADS-D data and are therefore not represented in the figure. HADS-D, Hospital Anxiety and Depression Scale depression subscale; POD, postoperative delirium.

### 3.3 Secondary outcome parameters

Linear regression assessing the effect of ODI (independent variable) on HADS-D (dependent variable) was statistically significant [F(1,85) = 20.36, *p* < 0.001, R^2^ = 0.19]. The regression coefficient (*β*) for ODI was 0.09 [95% CI [0.05, 0.13], t(85) = 4.51, *p* < 0.001]. Preoperative ODI scores showed no significant effect on the likelihood of developing postoperative delirium in the binomial logistic regression model. Analysis of the relationship between HADS-D and postoperative outcomes, including time to first mobilization and mean and median postoperative pain, revealed no statistically significant associations. In an exploratory secondary analysis, higher HADS-D scores were associated with greater peak postoperative pain [F(1,87) = 5.08, *p* = 0.03, R^2^ = 0.06]. The regression coefficient for HADS-D was 0.68 [95% CI 0.08–1.28; t(87) = 2.25, *p* = 0.03]. Participants with a HADS-D score ≥8 had higher peak postoperative pain ratings than participants with scores <8 (6.17 ± 1.43 vs. 4.94 ± 1.72; *p* = 0.01). HADS-D scores were not significantly associated with length of hospital stay.

## 4 Discussion

The present study examined whether preoperative depressive and anxiety symptom burden was associated with POD in older adults undergoing elective spine surgery. Higher preoperative depressive symptom scores were associated with greater odds of POD, both when analyzed continuously and using the established HADS-D threshold. In contrast, anxiety symptoms were not significantly associated with POD. Participants with a HADS-D score ≥8 had sixfold higher odds of POD than participants with scores <8. The continuous association remained significant after adjustment for age, sex, and preoperative cognitive performance. However, only 16 POD events were available for the complete-case HADS-D analyses, resulting in a limited event-to-parameter ratio and a risk of overfitting in the adjusted model; the adjusted estimate should therefore be interpreted cautiously. The binary association remained positive under extreme-case allocation of the six missing HADS-D values, but this sensitivity analysis does not eliminate potential selection bias in the continuous or adjusted analyses. Overall, these findings support preoperative depressive symptom burden as a potential marker of perioperative vulnerability but do not establish causality or demonstrate that treatment of depressive symptoms would reduce POD incidence.

These findings are consistent with previous studies reporting associations between depression and POD (21, 22). However, those analyses were largely retrospective and relied on psychiatric diagnoses documented in medical records, which may not capture subthreshold depressive symptom burden. In contrast, the present study used a validated symptom scale. One previous cohort also included patients receiving antidepressant medication, which may itself be relevant to delirium risk (21, 37). Another study reported an association between depression, impaired executive function, and POD across non-cardiac procedures (38). Direct comparison of effect estimates across studies is difficult because surgical populations, exposure definitions, and screening instruments differ. Higher ODI scores were associated with higher HADS-D scores in our cohort, whereas ODI was not significantly associated with POD. This reduces, but does not eliminate, concern that the observed HADS-D–POD association merely reflects back-pain-related disability; residual confounding remains possible.

The role of anxiety as an independent risk marker for POD remains uncertain. Similar to our findings, a previous study in older adults undergoing cardiac surgery reported no significant association between preoperative anxiety and POD (39). Given the limited number of participants with elevated HADS-A scores in the present cohort, however, the absence of a significant association should not be interpreted as evidence that anxiety has no effect on postoperative delirium risk.

In exploratory secondary analyses, higher HADS-D scores were associated with greater peak postoperative pain, but the model explained only 6% of the variance (R^2^ = 0.06). Moreover, analgesic medication was not systematically recorded. This finding should therefore be regarded as hypothesis-generating rather than as evidence of a clinically robust relationship. It is nevertheless consistent with previous reports linking preoperative affective symptoms with postoperative pain intensity (40).

The absence of an association between depressive symptoms and length of hospital stay in our cohort contrasts with findings reported by two previous studies (11, 12). Prior studies focused on clinically diagnosed major depression, whereas our analysis assessed depressive symptom burden using a screening instrument. Differences in exposure definition, patient population, and perioperative management may therefore partly account for the discrepant findings.

The clinical relevance of POD extends beyond the immediate postoperative period, as delirium has been associated with adverse longer-term cognitive outcomes (20). Depressive symptoms and POD may also share biological correlates, including systemic inflammation and altered stress-response signaling. Elevated Interleukin-6 has been associated with both depressive symptoms and postoperative delirium, while age-related inflammatory and vascular changes may reduce cognitive resilience under surgical stress (16, 41, 42). These mechanisms were not measured in the present study and are therefore presented only as possible biological context for the observed association, not as evidence of mediation or causality.

Postoperative delirium is a multifactorial outcome. In the parent CONFESS cohort, surgery duration, age, and deviations in intraoperative systolic blood pressure were associated with postoperative neurocognitive outcomes (24). These factors, together with other potentially relevant variables such as comorbidity burden, anesthetic exposure, intraoperative events, and postoperative medication use, were not comprehensively represented in the present adjusted model. Residual confounding therefore remains an important consideration, and the observed HADS-D association should be interpreted as complementary to established perioperative risk factors rather than as an isolated or independent determinant of POD.

Multicomponent delirium-prevention strategies—including orientation aids, sleep hygiene, early mobilization, and multimodal analgesia—have been evaluated in perioperative care as approaches to reduce delirium risk (43). In this context, brief preoperative HADS-D assessment could be considered as an additional component of geriatric risk assessment. A HADS-D score ≥8 identified a subgroup with higher observed POD odds in this cohort; however, sensitivity was only 50.0%, indicating that HADS-D should not be used as a stand-alone delirium screening or prediction tool. Rather, an elevated score may prompt closer evaluation of established delirium risk factors and consideration of enhanced multicomponent prevention measures. Whether interventions directed specifically at depressive symptoms can reduce POD requires prospective interventional testing.

### 4.1 Limitations

This study has several limitations. First, no separate *a priori* sample-size calculation was performed for this secondary analysis, and only 16 POD events were available for the complete-case HADS-D analyses. The adjusted logistic regression model therefore has a limited event-to-parameter ratio and is vulnerable to overfitting and imprecise effect estimation. Second, six patients who developed POD had declined the preoperative HADS assessment and therefore lacked HADS-D data. Missing HADS-D data were thus associated with outcome status, making the complete-case analyses susceptible to selection bias. In an extreme-case sensitivity analysis of the binary HADS-D comparison, the odds ratio ranged from 3.43 (95% CI 1.17–10.08) when all six missing POD cases were assigned to HADS-D < 8 to 10.50 (95% CI 3.57–30.86) when all six were assigned to HADS-D ≥ 8. Although the direction of the binary association was preserved under both extreme allocations, this analysis cannot eliminate potential bias in the continuous or adjusted models, for which the missing HADS-D values could not be recovered without unverifiable assumptions. Third, POD is multifactorial, and residual confounding by perioperative factors not included in the adjusted model remains possible. Fourth, despite repeated Nu-DESC screening and physician confirmation, short or fluctuating delirium episodes may have been missed, particularly during periods covered by chart review, potentially leading to underestimation of POD incidence. Fifth, analgesic medication was not systematically recorded, limiting interpretation of the exploratory postoperative pain findings; six participants also lacked complete ODI data. Finally, postoperative cognitive outcomes were not systematically assessed, precluding conclusions regarding long-term neurocognitive consequences. Generalizability may be limited by the single-center, procedure-specific cohort and the exclusion of patients with several neurologic or psychiatric conditions and centrally acting medications. Larger studies with prespecified sample-size planning, broader perioperative covariate capture, and structured neurocognitive follow-up are needed.

### 4.2 Conclusion

Higher preoperative depressive symptom burden was associated with POD in older adults undergoing elective spine surgery, whereas anxiety symptoms were not significantly associated. This association persisted after adjustment for age, sex, and preoperative cognitive performance but should be interpreted cautiously because of the limited number of POD events and residual confounding. Brief HADS-D assessment may complement, rather than replace, established perioperative geriatric risk assessment. Larger prospective studies are needed to validate the association and determine whether interventions targeting depressive symptoms can influence POD incidence.

## Data Availability

The raw data supporting the conclusions of this article will be made available by the authors, without undue reservation.
