# Supplementary material for: Preoperative depressive symptoms as a marker of postoperative delirium risk in older adults: a prospective cohort study in spine surgery
Source: Front Med (Lausanne). 2026 Sep 15;13:1936636. doi: 10.3389/fmed.2026.1936636 (PMC13619427; doi:10.3389/fmed.2026.1936636)
Supplement: Supplementary file 1 [file Supplementaryfile1.docx]

**Supplementary Figure S1**

Figure S1. Participant flow from eligibility screening to the final complete-case HADS-D analysis. Of 565 patients screened for study eligibility, 124 consented to participate in the CONFESS study. Twenty-five participants did not undergo the planned eligible surgery because consent was subsequently withdrawn or the planned procedure was cancelled, leaving 99 surgical participants. POD occurred in 22 of these 99 participants (22.2%). Six of the 22 patients who developed POD declined the preoperative HADS assessment and therefore had no HADS-D score available; they were excluded from HADS-D analyses. The final complete-case HADS-D cohort comprised 93 participants, including 16 with POD and 77 without POD. CONFESS, Cognitive Dysfunction Following Elective Spine Surgery in Older Patients; HADS-D, Hospital Anxiety and Depression Scale depression subscale; POD, postoperative delirium.
